# Effect of Electroacupuncture in Mice with Dextran Sulfate Sodium-Induced Colitis and the Influence of Gut Microbiota

**DOI:** 10.1155/2020/2087903

**Published:** 2020-04-27

**Authors:** Geng-Hao Liu, Hsuan-Miao Liu, Yu-Sheng Chen, Tzung-Yan Lee

**Affiliations:** ^1^Division of Acupuncture and Moxibustion, Department of Traditional Chinese Medicine, Chang Gung Memorial Hospital, Linkou, Taoyuan, Taiwan; ^2^Graduate Institute of Clinical Medical Sciences, College of Medicine, Chang Gung University, Taoyuan, Taiwan; ^3^School of Traditional Chinese Medicine, Chang Gung University, Taoyuan, Taiwan; ^4^Sleep Center, Chang Gung Memorial Hospital, Taoyuan, Taiwan; ^5^Graduate Institute of Traditional Chinese Medicine, College of Medicine, Chang Gung University, Taoyuan, Taiwan; ^6^Department of Traditional Chinese Medicine, Chang Gung Memorial Hospital, Keelung, Taiwan

## Abstract

**Background:**

The relationship between inflammatory bowel disease and gut microbiota is inextricable. Electroacupuncture (EA) can alleviate acute experimental colitis, but the performance of intestinal microorganisms and the mechanism are still not fully understood. We investigated the relationship between the EA and gut microbes and clarified the role of tight junction and adiponectin in the anti-inflammatory effect of EA.

**Methods:**

Male C57BL/6 mice were randomized into three groups: normal control, dextran sulfate sodium- (DSS-) induced ulcerative colitis (DSS), and DSS with EA ST36 (DSS + EA). Mice body weight, DAI score, colon length, and histological score were evaluated for colitis severity. Colonic inflammation and tight junctions were demonstrated by the immunohistochemical (IHC) method. Systemic responses were confirmed by plasma cytokines and adiponectin with multiplex immunoassays. Gut microbiome profiling was conducted by 16S rRNA gene sequencing.

**Results:**

EA had benefit in relieving both macroscopic and microscopic colonic inflammation. It can reduce disease activity, maintain colon length, and ameliorate histological inflammatory reaction. In IHC stain, EA decreased CD11b, F4/80, TLR4, and MyD88 and preserved claudin-1 and ZO-1 expression. Compared with the control group, the DSS group showed elevated levels of CRP, IFN-*γ*, TNF-*α*, and IL-6, but decreased adiponectin. These changes were reversed by EA, accompanied by modulation of the overall structure of gut microbiota.

**Conclusion:**

Our findings suggest that EA exerts its therapeutic effect by TLR4 signaling via the MyD88-dependent pathway. EA could increase adiponectin, maintain mucosal tight junctions, and modulate gut microbiota.

## 1. Introduction

Inflammatory bowel diseases (IBD), including ulcerative colitis (UC) and Crohn's disease (CD), result from chronic or relapsing immune activation and corresponding inflammation within the gastrointestinal tract. The prevalence of IBD in developed countries is more than in developing countries, which means not only genetic factors but also environmental factors are involved [1]. Lifestyle and diet habits [2] are closely related to immune regulation, and the newly defined “organ,” gut microbiota, seems to play a vital role in the pathophysiology. Besides, gut mucosal barrier dysfunction (leaky gut) and translocation of gut microbiota are possible mechanisms of IBD, and the tight junction is the first line of defense [3]; even till now there is no drug that could repair the tight junction in the gut mucosa.

Acupuncture therapy has been one of the popular complementary and alternative medicines for many chronic diseases, including inflammatory bowel disease. Because IBD is a chronic relapsing-remitting disease of the gastrointestinal tract, more and more people choose complementary and alternative medicine for the IBD adjuvant therapy in the western world [4]. Integrative medicine in IBD has been proven to increase the quality of life [5] and decrease the relapsing rate.

On the other hand, growing numbers of scientific research have indicated that acupuncture may be effective in treating many types of gastrointestinal diseases. Not only functional gastrointestinal disorders (FGID) [6] but also IBD has been rapidly mentioned in the past several decades [7]. Unlike FGID, acupuncture therapy plays an important role in gastroesophageal reflux disease (GERD) and functional dyspepsia (FD), but moxibustion treatment gets more attention in IBD clinical trial [8] and animal study [9].

Less but clear evidence of the mechanism of acupuncture/electroacupuncture (EA) in IBD includes cholinergic anti-inflammatory reflex [10], splanchnic sympathetic anti-inflammatory reflex [11], and hypothalamic-pituitary-adrenal axis which contributed to the anti-inflammatory effect [12, 13]. Other possible mechanisms, such as the opioid system [14], purinergic pathway [15], and macrophage polarization [16], were also discussed.

The purpose of this study is to investigate the efficacy of EA in IBD and to confirm the effect of EA in the tight junction in mice colitis model. Besides, we try to examine whether the neural-based EA treatment may modulate gut microbiota.

## 2. Materials and Methods

### 2.1. Animals

For the experiment, a total of 18 healthy male C57BL/6 mice weighing 20–25 g and aged 6–8 weeks were purchased from the National Laboratory Animal Center. The animals were reared in a controlled environment (22 ± 2°C and 50 ± 5% humidity) and under a 12 h/12 h light/dark cycle with *ad libitum* access to food and water.

This study was handled following the Guide for the Care and Use of Laboratory Animals. All experimental procedures were approved by the Chang Gung University Institutional Animal Care and Use Committee (IACUC Approval No. CGU15-070) and were performed with the least amount of animals used and animal manipulation.

### 2.2. Experimental Groups and Protocol

**Figure 1:**
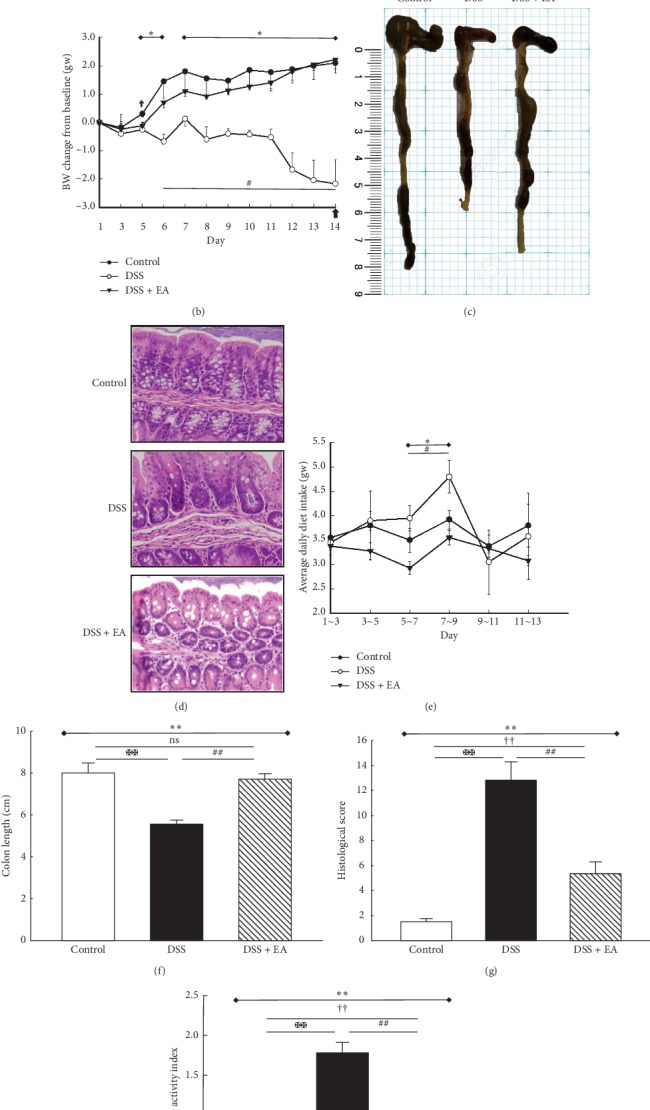
Electroacupuncture ameliorates dextran sulfate sodium- (DSS-) induced colitis in C57BL/6 mice. (a) Experimental protocol of 3.5% DSS colitis and electroacupuncture (EA) ST36 treatment course. (b) Mice body weights change from baseline after 3.5% DSS induction of colitis. (c) Intestine photograph of colorectum length in each group. (d) Representative H&E-stained colorectum sections (200x magnification) in mice with acute colitis. (e) Average daily chow diet consumption in each group. Statistics of (f) colon length, (g) histological score, and (h) disease activity index (DAI) in each group. ^*∗*^Results of three-group comparison (represented by bold line segments with endpoints). ^#^,^✠^, ^✟^Results of the DSS group versus DSS + EA group, DSS group versus control group, and DSS + EA group versus control group, respectively (represented as thin lines without endpoints). ^*∗*#✠†^*P* < 0.05; ^*∗∗*##✠✠††^*P* < 0.01; ns: no significance. Data were presented as mean ± SEM of six mice in each group.

Eighteen mice were divided into three groups as follows: (A) the untreated control group, (B) the group with dextran sulfate sodium- (DSS-) induced colitis, and (C) the group with DSS-induced colitis with electroacupuncture (EA) ST36 intervention. The experiment protocol is shown in Figure 1(a).

### 2.3. Induction of Experimental Colitis

Experimental colitis was induced by dextran sulfate sodium (DSS) (molecular weight: 6,500–10,000 Da; Sigma-Aldrich, St. Louis, MO, USA; 3.5%, added to the drinking water) for a total of 14-day experimental course, and DSS was changed every two days [17].

### 2.4. Electroacupuncture Intervention Procedure

From day 5 to day 13, EA ST36 was applied once per day to the DSS + EA group. The mice were kept in a supine position under anesthesia with 1.0–1.5% isoflurane inhalation to maintain the depth of anesthesia as stage III, which was evaluated by pedal reflex. Average body temperature was retained using warm thermal pads. The ST36 point in the mice is located at 5 mm lateral to and below the anterior tubercle of the tibia.

The area of acupoint was shaved and disinfected every time, and then a sterilized single-use stainless steel needle (0.27 mm in diameter and 13 mm in length; Ching-Ming Medical Co., Ltd., Taiwan) was inserted perpendicularly into both bilateral ST36 acupoints by a single experienced acupuncturist. The insertion depth was about 2-3 mm, which was marked as a red line on the body of the needle. After the *de qi* sensation, the EA stimulation was applied at both bilateral ST36 acupoints. An electroacupuncture apparatus (Digitimer DS3 stimulator, Letchworth Garden City, UK) was connected to the handles of both needles inserted at ST36 acupoints. EA was applied for 15 min, with an intensity of 1.0 mA and 2 Hz, associated with visible local muscle contraction.

### 2.5. Assessment of Colitis Severity

The mice colitis severity was assessed according to clinical score, colon length, and colon histological changes. Clinical evaluations, including body weight, stool consistency, and gross rectal bleeding, were recorded and scored to calculate the disease activity index (DAI) daily from day 1 to 14 at 10:00 a.m. [18]. Briefly, mouse weight was expressed as the relative change from day 1, and mouse weight loss of 1–5%, 5–10%, 10–20%, and >20% was scored as 1, 2, 3, and 4, respectively. Stool consistency was scored as 0 for normal well-formed pellets; 2 for loose, pasty, and semiformed stools, which did not adhere to the anus; and 4 for diarrhea, which means liquid stools that adhere to the anus. For rectal bleeding, a score of 0 was given for no blood in stools, 2 for gross bleeding from the anus, and 4 for gross bleeding in stools. The DAI was expressed as the following equation: DAI = (body weight loss + stool consistency + gross rectal bleeding)/3 [19].

The animals were sacrificed by decapitation under anesthesia. The colon was excised immediately between the ileocecal junction and the anus, and its length was determined with a ruler. Later, the small pieces of the colon tissues were fixed in 4% paraformaldehyde in PBS, embedded in paraffin, and sliced into sections of 5 *μ*m thickness for histological examination. After H&E staining, histological changes were observed under a light microscope, and the immunohistochemical studies were performed on this section afterward.

The determination of the histopathology score for intestinal inflammation of the colon was performed using a validated scoring system [20]. According to the percentage of the six items, namely, goblet cell loss, mucosal thickening, inflammation cells, submucosal cell infiltration, destruction architecture, and ulcers, scores 0, 1, 2, 3, and 4 mean 0, 0–25%, 25–50%, 50–75%, and 75–100%, respectively.

### 2.6. Immunohistochemistry Stain

To evaluate the local inflammatory components and pathways, the expressions of the monoclonal antibodies used to characterize murine macrophages (CD11b, F4/80, both bought from Abcam Inc., USA), the toll-like receptors (TLR4, bought from Abcam Inc., USA), and the classical pathway (MyD88, bought from Chemicon International, Inc., USA) were detected in the colon tissue by the immunohistochemical method (IHC stain). Besides, to evaluate the gut barrier function, the presentations of the tight junction proteins (claudin-1, ZO-1, both bought from Abcam Inc., USA) were also recognized through the same way as IHC stain was performed on the blank section preprocessed from colonic tissues. The display of CD11b, F4/80, TLR4, MyD88, claudin-1, and ZO-1 was presented by histologic sections at 200x magnification. Images of IHC slides were quantified by calculating the percentage of DAB stained area through true color image analysis with the application of adjusted thresholds in ImageJ.

### 2.7. Plasma Biomarker Analysis

In the analysis of plasma biomarkers for inflammatory response and adiponectin, plasma was isolated from trunk blood collected from the site where the animal was decapitated under CO_2_ anesthesia at the time of euthanasia and aliquoted for storage at −30°C until further use. The plasma concentrations of the proinflammatory cytokine (IFN-*γ*, TNF-*α*, and IL-6) and the C-reactive protein (CRP), as well as the adiponectin, were quantified using multiplex immunoassay technology xMAP on a Luminex® 200™ instrument (Luminex) according to manufacturer's instructions. Mouse plasma was used without dilution for analysis of IFN-*γ*, TNF-*α*, and IL-6 using a custom-designed mouse multiplex assay kit (IFN-*γ*, EPX01A-20606-901; TNF-*α*, EPX01A-20607-901; IL-6, EPX01A-20603-901; ProcartaPlex™ assays; Thermo Fisher Scientific, Waltham, MA, USA). Plasma was diluted 1 : 10000 for CRP analysis using a mouse ProcartaPlex™ Simplex Kit (EPX01A-26045-901; Thermo Fisher Scientific) and 1 : 2000 for adiponectin analysis using a mouse ProcartaPlex™ Simplex Kit (EPX01A-26038-901; Thermo Fisher Scientific). The quantification of the lower limit of detection (LLOD) using this system was 1.1, 3.4, 5.3, 34.0, and 36.6 pg/ml for IFN-*γ*, TNF-*α*, IL-6, CRP, and adiponectin, respectively. Samples with values below the lowest limit of detection (LLOD) were assigned a value of half the LLOD for statistical purposes.

### 2.8. Stool Collection, Gut Microbiota Processing, and 16S Metagenomics Analysis

The excised colon was opened longitudinally, and the stool pellets were collected in the fume hood, followed by microbiota processing and analysis.

Fecal DNA extraction and 16S rRNA gene sequencing procedure were performed by the Genomics BioSci & Tech. Co., Ltd. (Taipei, Taiwan). Briefly, 1 *μ*l of sample DNA (10 pg∼500 ng) was used as a template in a PCR reaction for bacteria 16S rRNA variable region V3∼V4 [21]. The specific primer sets for the reaction were 341F (V3_Illumina 5′-CCTACGGGNGGCWGCAG-3′) and 805R (V4_Illumina 5′-GACTACHVGGGTATCTAATCC-3′). The PCR amplified 400∼500 bp fragments were excised in the process of gel extraction and purified using QIAquick Gel Extraction Kit (Qiagen). TruSeq DNA Sample Preparation Kit (Illumina) was used for the library construction, and then the prepared library was quantified using TruSeq Nano DNA Sample Preparation Kit (Illumina) before loading on to the up-to-date NGS sequencer, MiSeq (Illumina). The sequences of 2 × 300 bp paired-end reads were produced from the sequencer following the instructions of the manufacturer.

Due to the enormous amount of data, more than one million read pairs, the paired reads would be merged into amplicon sequences; then, these amplicon sequences were checked for the existence of the primers, removing the duplicates and filtering out short sequences, and finally clustered into OTUs (Operational Taxonomic Units). These steps reduced the number of amplicon sequences and grouped them into represented OTUs for further analysis, including 16S rRNA database searching and taxonomic assignments, by using the bioinformatics software package “mothur” v. 1.33.3 and “QIIME” v. 1.8.0 [22].

All valid reads were calculated for pairwise distances between aligned DNA sequences with cutoff as 0.03 and then clustered into OTUs using “average neighbor algorithm” with hard cutoff as 0.03; these final classified OTUs were aligned against Greengenes 16S rRNA Taxonomy Database (gg_13_8) and then assigned taxonomy on different taxonomic ranks: kingdom, phylum, class, order, family, genus, and species.

The representative sequences of OTUs and their relative abundance were used to calculate the alpha diversity, including rarefaction analysis (Chao1 stands for community richness, and Shannon stands for community diversity), rank abundance, and Venn diagram. Beta diversity (principal component analysis (PCA), phylogenetic tree) was then used to analyze the diversity between groups.

### 2.9. Statistical Analysis

Statistical analysis was conducted using IBM SPSS Statistics 21.0 Software (IBM Corp., released 2012, IBM SPSS Statistics for Windows, Version 21.0; Armonk, NY). All data were analyzed using the Kruskal–Wallis ANOVA test and presented as mean ± SEM. The Mann–Whitney *U* test was employed to determine the difference between every two groups. *P* values <0.05 were considered statistically significant.

## 3. Results

### 3.1. EA ST36 Generates Therapeutic Effect in the Mice UC Model

The most frequent clinical index and golden macroscopic evidence in IBD are body weight loss and shortened colon length, respectively. Thus, the core strategy was to evaluate the average daily body weight change under 3.5% DSS treatment, followed by measuring the colon length at the time of scarification. As indicated in Figure 1(b), the DSS group exhibited a significant reduction in daily body weight change (*P* < 0.05), which was significantly remedied by treatment with EA ST36 (*P* < 0.05). With the daily diet intake in Figure 1(e), no significant difference was noted among the three groups, except that DSS group had an increased diet intake during day 7 to 9. Taken together, even DSS group had the same or more diet intake, and the mean body weight change was dropped significantly when compared to control group and DSS + EA group. Also, macroscopic finding in Figures 1(c) and 1(f) showed that colon length in DSS group (5.53 ± 0.56 cm) was obviously shortened compared to control group (8.00 ± 1.09 cm) and DSS + EA group (7.71 ± 0.65 cm) (*P* < 0.05), and microscopic finding in Figures 1(d) and 1(g) demonstrated loss of goblet cells and infiltrated inflammatory cells, accompanied by increased histopathology score (12.83 ± 3.54 in DSS group vs. 1.50 ± 0.55 in control group and 5.33 ± 2.34 in DSS + EA group). Finally, when considering the rectal bleeding and diarrhea as major clinical symptoms, disease activity index (DAI) in Figure 1(h) showed the same tendency; the DSS group was the highest (1.78 ± 0.35), followed by the DSS + EA group (0.73 ± 0.13) and control group (0.17 ± 0.18). In summary, EA ST36 could maintain colon length, reduce disease activity, and ameliorate histological inflammatory reaction.

### 3.2. EA ST36 Attenuates Colonic Inflammatory Response and Ameliorates Epithelial Tight Junctions

#### 3.2.1. EA ST36 Diminishes Macrophages Infiltration in Bowel Inflammation

**Figure 2:**
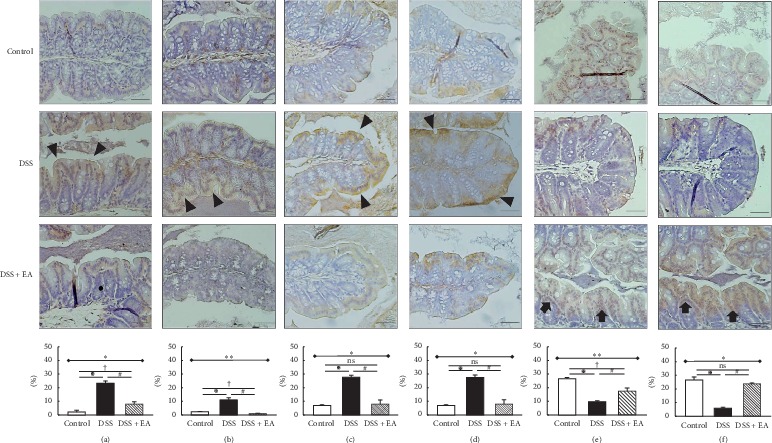
Immunohistochemical (IHC) staining of colon sections (200x magnification). Upper part: expression of (a) CD11b, (b) F4/80, (c) TLR4, (d) MyD88, (e) claudin-1, and (f) ZO-1 antibody in colon tissue of different groups. The arrowheads in the DSS group indicate the positive yellow and brown staining for these proteins, which mean that macrophages infiltrated bowel inflammation (a, b) through TLR4 signaling via MyD88-dependent pathway (c, d). The arrows in DSS + EA group indicate the positive brown staining for these proteins, which mean tight junction preservation (e, f) when compared to the DSS group. Scale bar: 50 *μ*m. TLR4: Toll-like receptor 4. EA: electroacupuncture ST36. Lower part: quantification of area percentage of IHC staining by true color image analysis with the application of adjusted thresholds. ^*∗*^Results of three-group comparison (represented by bold line segments with endpoints). ^#^, ^✠^, ^✟^Results of the DSS group versus DSS + EA group, DSS group versus control group, and DSS + EA group versus control group, respectively (represented as thin lines without endpoints). ^*∗*#✠†^*P* < 0.05; ^*∗∗*^*P* < 0.01; ns: no significance. Data were presented as mean ± SEM of repeated adjusted thresholds in each group.

DSS colitis is a chemical-induced colitis model; we performed an immunohistochemical (IHC) analysis to determine the composition of leukocytes present in the colon on day 14. In Figures 2(a) and 2(b), a considerable number of macrophages were already found in the lamina propria of the DSS group, demonstrated by the apparent presentation of CD11b and F4/80 in the DSS group. Besides, IHC in Figures 2(c) and 2(d) revealed marked tint of TLR4 and MyD88 in DSS group but not in control or DSS + EA groups. Taken together, EA ST36 may act in part through TLR4 signaling via MyD88-dependent pathway to improve colonic inflammation.

#### 3.2.2. EA ST36 Preserves the Epithelial Tight Junctions

DSS disrupts intestinal barrier function, which is the predisease stage in IBD. The following translocation of microbial products is a core cause of further immune cell activation and intestinal inflammation. In Figures 2(e) and 2(f), IHC stain revealed distinct tight junction proteins, both claudin-1 and ZO-1, in the DSS + EA group, instead of becoming fuzzy in the DSS group. This means that EA ST36 could retain the tight junctions in DSS colitis model.

#### 3.2.3. EA ST36 Relieves Systemic Inflammatory Response and Recovers the Adiponectin Level

In addition to local macroscopic and microscopic inflammatory manifestations of the intestine, we also confirmed systemic inflammatory response through plasma. CRP, IFN-*γ*, TNF-*α*, and IL-6 were all elevated in the DSS group significantly. Among them, EA could significantly improve the rising value in the CRP and IFN-*γ* levels (compared to the DSS group, both *P* values = 0.004). For TNF-*α* and IL-6, the EA group also showed a downward trend. TNF-*α* decreased from 3.04 to 2.29 pg/ml, and IL-6 decreased from 26.08 to 10.83 pg/ml. On the other hand, EA could significantly increase the adiponectin level that decreased after DSS administration to a value close to that of the control group. *P* values were both 0.004 in comparison with DSS versus control group and DSS versus EA group.

### 3.3. Influence of EA ST36 on the Gut Microbiota

#### 3.3.1. EA ST36 Tends to Modulate the Overall Structure of Gut Microbiota

**Figure 3:**
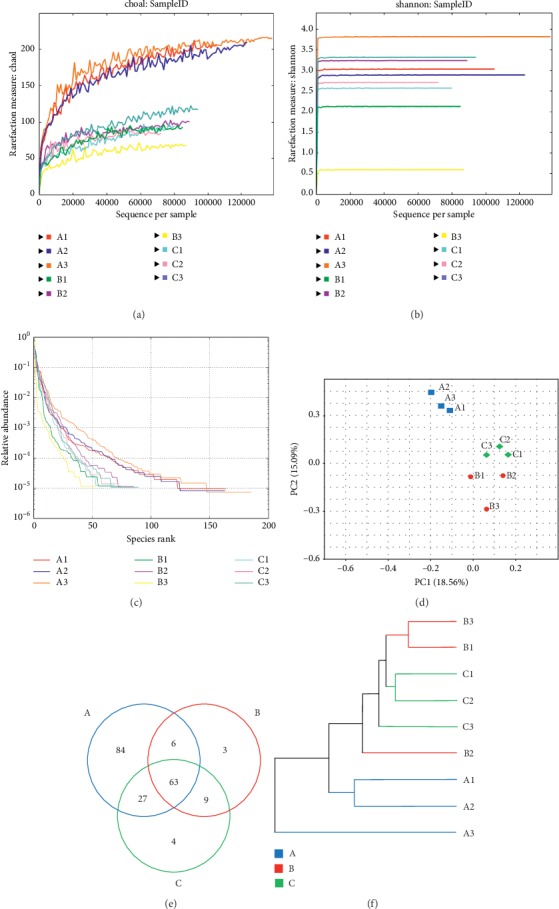
Overall structural modulation of gut microbiota after EA ST36 treatment. (a) Rarefaction analysis on Chao1 curves for nine samples, a measure of species richness. (b) Rarefaction analysis on Shannon curves for nine samples, a measure of species diversity. (c) The rank abundance of samples. (d) Multiple-sample PCA. (e) Venn diagram of OTUs in the three groups. (f) Multiple-sample similarity tree. (A) Samples of the control group, (B) samples of the DSS group, (C) samples of the DSS + EA group (a–f, *n* = 3). EA: electroacupuncture ST36.

Half the samples of each group were sent for intestinal microbiota processing and 16S metagenomics analysis. When getting quality-filtering and nonchimeric data, we used mothur to assign each OTU to the possible bacterial taxonomic category. In a total of nine fecal samples, each sample was normalized to equal sequencing depth and clustering. After filtering the low abundant (<0.1%), 196 OTUs were obtained at ≥97% sequence identity. As shown in Figures 3(a) and 3(b), gut dysbiosis could be identified when comparing DSS group to control group, but the richness and diversity of rarefaction analysis showed no significant differences between DSS and DSS + EA groups at the OTU level. A similar result could be found in Figure 3(c). Rank abundance curves demonstrated that A1–A3, standing for fecal samples from the control group, were separated into another group away from B and C fecal samples. However, the Venn diagram showed a significant modulation of OTUs (*P* = 0.027) among the three groups. In Figure 3(e), the overlap of OTUs among groups revealed that 63 OTUs coexisted in all the three groups. Further 69 OTUs were present in both the control and DSS groups, 72 in the DSS and DSS + EA groups and 90 in the control and DSS + EA groups. PCA (Figure 3(d)) revealed that the gut microbiota in the DSS group deviated from the baseline structure in the control group, and the DSS + EA group became another cluster gradually but did not return to the level of the control group. The multiple-sample similarity tree illustrated that the level of the DSS + EA group was close to that of the control group (Figure 3(f)).

#### 3.3.2. EA ST36 Regulates Structural Segregation of Gut Microbiota in Mice

**Figure 4:**
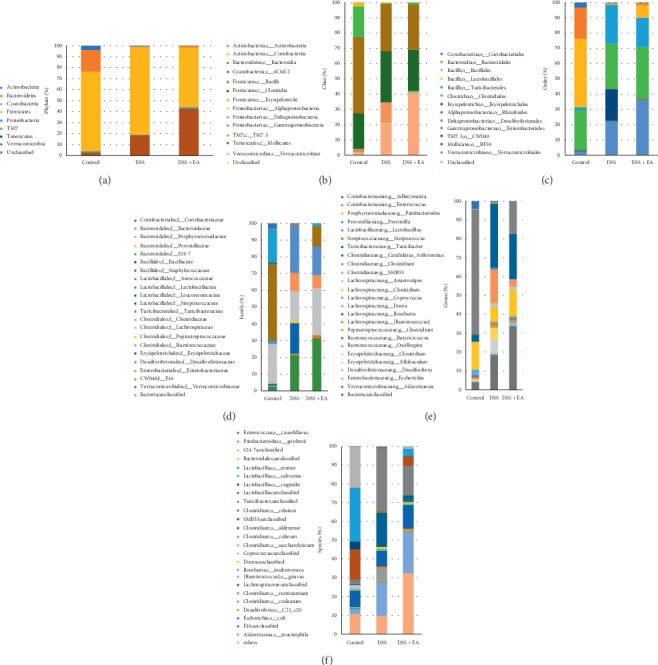
Gut microbial community structure in mice after EA treatment. (a) Microbial community bar plot by phylum. (b) Microbial community bar plot by class. (c) Microbial community bar plot by order. (d) Microbial community bar plot by family. (e) Microbial community bar plot by genus. (f) Microbial community bar plot by species. From left to right, control, DSS, and DSS + EA groups, respectively. *N* = 3 in each group. EA: electroacupuncture ST36.

Histograms illustrating the gut microbiota community structure reveal the microbial species and their relative abundance. As shown in Figure 4(a), the most abundant phyla in the control group were Firmicutes and Bacteroidetes, and the top two most phyla are Firmicutes and Verrucomicrobia in both DSS and DSS + EA groups. These three phyla and TM7 meet the significant differences of the relative abundance of gut microbiota among the three groups at the phylum level (upper part of Table 1). Among the four phyla, only Firmicutes had significant difference between DSS and DSS + EA group (*P*=0.016). At the class level displayed in Figure 4(b), Coriobacteriia, Bacteroidia, Erysipelotrichi, Gammaproteobacteria, TM7-3, and Verrucomicrobia were significantly different among the three groups, and only Erysipelotrichi had significant difference between the DSS and DSS + EA groups (*P*=0.019).

At the order level illustrated in Figure 4(c), Bacteroidales (*P*=0.012) and Lactobacillales (*P*=0.006) were dominant in control group, had decreased percentage in DSS group, then restored percentage in DSS + EA group. On the flip side, Turicibacterales and Erysipelotrichales were found in tiny proportions in control group, but much elevated in DSS group, and obviously cut back in DSS + EA group (*P*=0.007, *P*=0.013, respectively). At the family level demonstrated in Figure 4(d), the trend continues at the order level, S24-7 (*P*=0.002) and Lactobacillaceae (*P*=0.006) were detected in the control group but were rare in DSS group; then they were elevated in DSS + EA group. On the other hand, Turicibacteraceae, Clostridiaceae, and Erysipelotrichaceae were found in scant percentages in control group, but the percentage moved upwards in DSS group and downwards in DSS + EA group (*P*=0.007, 0.029,  and 0.006, respectively). Going to the genus level (Figure 4(e)), excluding some genera that changed significantly but are unclassified (e.g., S24-7), *Lactobacillus*, *Turicibacter*, and SMB53 were three genera that had significant difference among the three groups. Among them, *Lactobacillus* was found at significantly lower levels in the DSS group compared with those in the control and DSS + EA group (*P*=0.004). Conversely, the relative abundance of *Turicibacter* (*P*=0.007) and SMB53 (*P*=0.003) was significantly higher in the DSS group compared with the control and DSS + EA group.

#### 3.3.3. EA ST36 Tends to Regulate the Growth of Certain Bacteria

In the gut flora analysis at the species level (Figure 4(f)), some identified specific bacteria had significant difference among the three groups, including *Lactobacillus reuteri*, *Lactobacillus vaginalis*, *Clostridium ruminantium*, and *Akkermansia muciniphila* (lower part of Table 1). Among these four bacteria, *Lactobacillus reuteri* and *Lactobacillus vaginalis* had higher abundance percentage in the control group, but it dropped down in the DSS group and then slightly regained their percentage in the DSS + EA group without statistical significance (*P*=0.056 and 0.833, separately). *Clostridium ruminantium* was identified in the DSS and DSS + EA groups (*P*=1.000 in these two groups) but was not detected in the control group. In the experiment, *Akkermansia muciniphila* was very special because it became the dominant microorganism in both DSS and DSS + EA groups since its phylum level is Verrucomicrobia. Besides, its proportion even increased after EA intervention (*P*=0.527 when comparing DSS group to DSS + EA group).

## 4. Discussion

This study has illustrated that repeated EA treatment may generate a therapeutic effect in mice UC model, attenuate colonic inflammatory response, and have influences on gut microbiota. TLR4/MyD88 pathway and amelioration of the epithelial tight junctions might partly explain the beneficial effect of EA on colitis.

**Figure 5:**
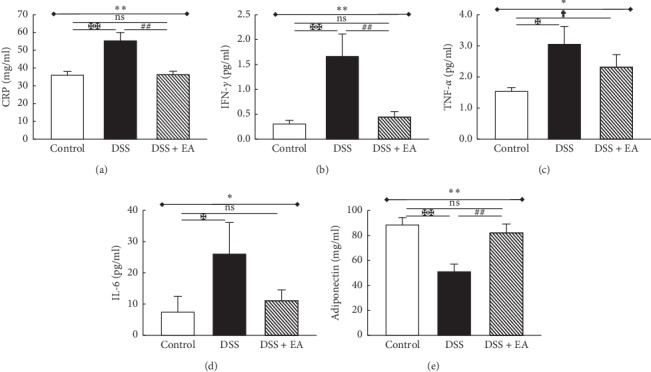
Electroacupuncture decreases plasma inflammatory marker/cytokines and increases adiponectin in dextran sulfate sodium- (DSS-) induced colitis in C57BL/6 mice. (a) C-reactive protein (CRP), as an inflammatory biomarker, was elevated in the DSS group and declined in DSS + EA group. Similar trends were observed in other proinflammatory cytokines, including (b) IFN-*γ*, (c) TNF-*α*, and (d) IL-6. (e) Adiponectin was decreased in DSS group and increased in DSS + EA group. ^*∗*^Results of three-group comparison (represented by bold line segments with endpoints). ^#^, ^✠^, ^✟^Results of the DSS group versus DSS + EA group, DSS group versus control group, and DSS + EA group versus control group, respectively (represented as thin lines without endpoints). ^*∗*#✠†^*P* < 0.05; ^*∗∗*##✠✠^*P* < 0.01; ns: no significance. Data were presented as mean ± SEM of six mice in each group.

We assessed the severity of colitis after EA treatment, whether the clinical disease activity index, macroscopic colon length, or microscopic histopathologic findings have been significantly improved. In clinical evaluation, body weight was an important indicator for mice UC model, and the body weight change from baseline provided another clear evidence of the effect of EA treatment. At the same time, we recorded the daily diet intake in each group, to clarify the effect of food consumption on body weight changes. EA ST36 has been proven to change food intake in different conditions. In high-fat-diet-induced obese mice, EA ST36 could inhibit food intake and reduce body weight [23]. However, in chronic psychological stress (CPS) rats [24] or rodent model of cisplatin-induced dyspepsia [25], EA ST36 could improve food intake and weight. It seems that ST36 makes modulation of diet intake, whether by sympathetic nerve activation or vagal stimulation, and can achieve the purpose of attenuating the inflammatory response. In DSS-induced colitis mice, the food intake of the EA ST36 group was less than that of the DSS group and the control group. Therefore, the body weight of the EA group can be maintained because of the anti-inflammatory effect, which was demonstrated in Figures 2 and 5, rather than merely increasing the food intake.

Although the exact etiology of IBD remains elucidated, data from many animal models and human studies imply that it is associated with the destruction of intestinal epithelial integrity, causing colon injury and leading to the translocation of intestinal microbes. The host-microorganism interaction is accompanied by the presence of inflammatory cells and increased levels of proinflammatory cytokines, including CRP, IFN-*γ*, TNF-*α*, and IL-6. CRP, which is an acute-phase protein stimulated by infection, inflammatory diseases, tissue infarction, and neoplasia, is one of the essential molecules in host defense and regulation of cascaded inflammatory cytokines and monocyte-macrophage series. IFN-*γ* and TNF-*α* are both central pathogenesis factors in IBD with pleiotropic effects on many different cell types. IFN-*γ* is related to increased vascular permeability and endogenous angiostatic activity in IBD [26], and TNF-*α* has the function of modulating intestinal mucus secretion and constitution [27], which impacts epithelial wound healing in the regulation of the intestinal epithelial response to inflammation. Both are critical parts of the complex process of intestinal homeostasis. IL-6 is generated by mononuclear macrophages, fibroblasts, endothelial cells, T cells, and B cells, which possess various biological properties. It is overexpressed in inflammatory bowel mucosa, associated with IL-1, IL-17, and TNF-*α*, leading to the occurrence of inflammation in UC. In the present study, it was observed that the levels of CRP, IFN-*γ*, TNF-*α*, and IL-6 were elevated in mice plasma with DSS-induced colitis. The administration of EA ST36 significantly suppressed the levels of proinflammatory mediators in DSS-induced colitis mice model.

Intestinal epithelial cells (IECs) create various mucosal barriers to separate the intestinal flora and intestinal immune cells to prevent excessive immune responses that cause intestinal inflammation. During the process, IECs express a variety of pattern recognition receptors, including Toll-like receptors (TLRs), to directly detect bacterial constituents. Antibacterial molecules produced by IECs are controlled by intestinal microorganism-driven TLR4/MyD88 signals [28]. In the healthy intestine, TLR4 is present only in small amounts on IEC and lamina propria mononuclear cells (LPMNCs) in vivo. TLR inhibition acts to avoid inappropriate activation despite the omnipresent microbiota. Once host threats are encountered, these inhibitory mechanisms can be switched off, and positive regulators allow TLR signaling to elicit critical immune responses, in an attempt to eliminate the danger. However, sustained TLR hyperactivation may provoke chronic inflammation in IBD [29]. EA at Zusanli (ST36) acupoint exerts anti-inflammatory effects on the TLR4 signaling pathway in many murine models, including cerebral ischemia-reperfusion injured rats [30], adjuvant arthritis rats [31], or traumatic brain injury rats [32]. The crucial signaling molecules in the TLR4/NF-*κ*B signaling pathway were regulated by acupuncture, which coincided with suppressed secretion levels of inflammatory cytokines such as TNF-*α*, IL-1*β*, and IL-6. In our results, we demonstrated that repeated EA intervention ameliorates DSS-induced colitis by suppressing proinflammatory mediators, including CRP, IFN-*γ*, TNF-*α*, and IL-6 through the TLR4 signaling via MyD88-dependent pathway.

Acute dextran sulfate sodium- (DSS‐) induced colitis has been shown to promote gut microbial dysbiosis in mice, including reducing bacterial species richness and altering bacterial community composition [33]. As for acupuncture studies, although some animal experiments showed that EA was beneficial in relieving the symptoms and signs of UC with partial restorative effect on gut flora [34], such as decreased enteric *Clostridium bifermentans* content and increased enteric Lachnospiraceae bacterium and *Lactobacillus* sp., most of the experiments were designed to stop or reduce the administration of DSS after successful modeling. This means that few studies were designed to simulate the real clinical situation; that is, the large intestine is a persisted inflammatory ecosystem. In our study, we used EA ST36 intervention in the continuously inflamed colon, in order to avoid the factors of self-healing of the intestine, which may interfere with the evaluation of the efficacy of EA, and further to clarify whether the anti-inflammatory effect of EA plays an important role in intestinal epithelial homeostasis. The result proves that EA not only could maintain the colon length and daily body weight, but also could modulate the overall structure of gut microbiota in PCA, Venn diagram, and similarity tree, even with the DSS administration throughout the whole process. As for the specific species, *Lactobacillus* could be decreased by DSS administration, which matched our data, and it could be reversed after EA intervention.

Dysbiosis in the animal colitis model has been confirmed to be associated with intestinal inflammation. However, the loss of tight junction proteins, such as ZO-1/claudins, and increased permeability preceded the development of severe bowel inflammation, suggesting that in DSS colitis alterations in the tight junction complex occur before the intestinal inflammation and not as a consequence of it [35]. Tight junctions, together with adherens junctions, construct the apical junctional complex, so it can control epithelial permeability and further maintain intestinal homeostasis. EA has been proposed to improve the gut mucosal barrier function in circulatory and infectious conditions, which were animal models of hemorrhagic shock [36] and sepsis [37]. In a rat model of CLP-induced sepsis, electrical stimulation of ST36 can increase protein expression of occluding, improve the intestinal damage index, and protect the immune barrier of the intestinal mucosa. These results can be observed even in our model of intestinal inflammation. In addition, EA ST36 could not only protect intestinal barrier integrity, but also attenuate the systemic inflammatory response and improve organ function and survival rate in animals with hemorrhagic shock, by activating the cholinergic anti-inflammatory mechanism. In our findings, we further confirmed that EA might have other effects besides the cholinergic anti-inflammatory mechanism, namely, the TLR4 and MyD88 signaling pathway, which is another possible way highly related to the modulation of gut immunity.

Adiponectin, secreted by mesenteric adipose tissue, has had very different results in intestinal inflammation, but recent studies have shown that it has a critical effect on the intestinal epithelium; it could not only inhibit macrophages infiltration and proinflammatory cytokines release [38], but also maintain intestinal homeostasis and improve intestinal barrier integrity [39]. In some mice DSS models, adiponectin has been confirmed to enhance colonic expressions of tight junctions (ZO-1 and claudin-1) after DSS exposure. These three mechanisms can be confirmed in our results of EA treatment, and EA ST36 also does show a recovery in adiponectin performance during DSS management. Hence, it may provide EA with another possible mechanism in modulation of dysbiosis in DSS mice.

The major limitation of our study was that the numbers of fecal samples were too small due to limited budget. A closer look at the analysis of intestinal microbes also reveals that a large proportion of microbiota were unclassified bacteria. It may be because the previous microbial comparison database has not been updated in time. The unknown strain still has an eternal existence like the ocean. We already know a lot, but more are still unknown, which depends on more research on intestinal microorganisms. Another frequently asked question is whether the findings from mice can be applied to humans. We know that creeping fat is a phenomenon found in patients with Crohn's disease and ulcerative colitis, but it is absent in the DSS model of colitis [40]. Therefore, even mesenteric adipose tissue (MAT) is thought to form a reactive immunological zone around the inflamed intestine, but it is not easy to clarify the function of MAT especially in DSS mice.

## 5. Conclusions

In conclusion, there are convincing results about EA in terms of colon length and daily body weight change in the mice colitis model, even with the DSS administration throughout the whole process. Not surprisingly, in this model of acute colitis, the overall structure of the intestinal flora was adjusted as the degree of intestinal inflammation improved. Tight junctions may be the crucial part of the mechanism in EA-treated DSS colitis in mice, and adiponectin possibly has a role in the epithelial hemostasis and anti-inflammation effect.

## Figures and Tables

**Table 1:** Significant differences in the relative abundance of gut microbiota among the three groups at the phylum level and the species level (relative abundance is <0.1% or unclassified species were not presented). # represents the results of DSS group versus DSS + EA group having *P* value of <0.05.

	Control	DSS	DSS + EA	*P* value
Phylum level				
Bacteroidetes	18.76 ± 6.59%	0.56 ± 0.33%	1.32 ± 0.80%	0.008^*∗∗*^
Firmicutes	69.14 ± 7.28%	88.84 ± 3.83%	54.20 ± 13.31%^#^	0.018^*∗*^
TM7	0.26 ± 0.13%	0.01 ± 0.01%	0.04 ± 0.04%	0.039^*∗*^
Verrucomicrobia	2.62 ± 1.50%	20.65 ± 5.94%	42.60 ± 14.62%	0.006^*∗∗*^

Species level				
*Lactobacillus reuteri*	28.40 ± 6.75%	0.27 ± 0.10%	3.80 ± 0.80%	0.004^*∗∗*^
*Lactobacillus vaginalis*	4.36 ± 1.05%	0.18 ± 0.13%	0.28 ± 0.18%	0.004^*∗∗*^
*Clostridium ruminantium*	0.00 ± 0.00%	0.80 ± 0.39%	0.34 ± 0.22%	0.04^*∗*^
*Akkermansia muciniphila*	2.60 ± 1.49%	18.27 ± 6.50%	22.18 ± 8.98%	0.039^*∗*^

## Data Availability

The data used to support the findings of this study are available from the corresponding author upon request.
